# Baclofen as relapse prevention in the treatment of Gamma- Hydroxybutyrate (GHB) dependence: an open label study

**DOI:** 10.1186/s12888-015-0471-4

**Published:** 2015-04-28

**Authors:** Rama M Kamal, Arnt Schellekens, Cornelis AJ De Jong, Boukje AG Dijkstra

**Affiliations:** Nijmegen Institute for Scientist-Practitioners in Addiction (NISPA), Hogedwarsstraat 3, PO Box 243, Vught, 5260 AE the Netherlands; Novadic-Kentron Addiction Care network, Vught, the Netherlands

**Keywords:** Baclofen, Gamma- Hydroxybutyrate, GHB dependence, Relapse, Craving

## Abstract

**Background:**

GHB dependence is a growing health problem in several western countries, especially the Netherlands. Attempts to stop using GHB are often followed by relapse shortly after successful detoxification. Craving for GHB use and co-morbid psychiatric symptom levels are thought to be the major factors contributing to the high relapse rates. Given its pharmacological profile, baclofen might prove an effective anti-craving agent for patients with GHB dependence. The aim of the current study is to assess the potential of baclofen as an anti-craving agent relapse prevention intervention in GHB dependent patients.

**Methods/Design:**

In an open label non-randomized trial treatment with baclofen to a maximum of 60 mg/day will be compared with treatment as usual (TAU) in recently detoxified GHB dependent patients (n = 80). The primary outcome measure will be the level of GHB use. Secondary outcome measures are craving levels, psychiatric symptom levels and quality of life. Questionnaires will be administered during 12 weeks of baclofen treatment and at follow-up (six months after the start of treatment).

**Discussion:**

It is hypothesized that baclofen treatment compared to TAU will be associated with significantly reduced GHB use. In addition, we hypothesize that baclofen treatment will be associated with decreased craving and anxiety levels, and higher quality of life. If results are in line with our hypotheses, further studies on the efficacy of baclofen using placebo controlled designs and long term follow-up are warranted.

**Trial registration:**

The Netherlands Trial Register with number NTR4528. Registered 19 April 2014.

## Background

GHB use is a growing public health issue in several Western countries, including the Netherlands [1]. Recreational use of GHB has gained popularity over the past decades [2]. As a result, it’s addictive potential has become more apparent [3]. Little is known about the exact prevalence of chronic GHB dependence in the USA and Europe due to the absence of surveillance and systematic reporting mechanisms [4]. Nevertheless the number of GHB users seeking help increased over the past years [5]. For example, the number of GHB dependent patients admitted in addiction treatment facilities sharply increased in the Netherlands, over the last five years from 60 in 2008 to almost 800 patients in 2013 [6].

Gamma-hydroxybutyrate (GHB) is a short-chain fatty acid that is an endogenous precursor and metabolite of gamma-aminobutyric acid (GABA). GHB administered systemically can cross the blood–brain barrier where it acts both as neurotransmitter and a neuromodulator [7]. It has a plasma half-life of approximately 30–60 minutes [8]. GHB has high affinity for the GABA-B receptor and to a lesser extent for subtypes of the GABA-A receptor [9]. GHB has impact as neuromodulator via both GABA-ergic effects and direct effects on a wide variety of other neurotransmitters, including glutamate, dopamine, serotonin, noradrenaline, acetylcholine, opioids, and GABA [10-12]. GHB has various therapeutic applications, like general anesthesia [13], treatment of sleep disorders as narcolepsy [14], and the treatment of alcohol [15] and opioid withdrawal [16].

GHB tolerance occurs rapidly when used daily, inducing physical dependence at higher doses. Discontinuation of GHB can produce severe withdrawal symptoms as anxiety, delirium with auditory and visual hallucinations, seizers, and coma, which may be life threatening [17,18]. Recent studies have shown safety of strategies for the detoxification in GHB dependence, using tapering with pharmaceutical GHB or benzodiazepines [19]. Nevertheless, high relapse rates hamper long-term recovery, despite psychological treatment and counseling. Forty-five percent of the cases reporting to addiction care facilities had previously been in treatment for GHB dependence [19]. Indeed, short-term relapse rates up to 64% have been reported [20]. Self-reported reasons for relapse include social pressure, craving for and loss of control over GHB use and increased anxiety and depression after stopping GHB use [21].

Here we present a study protocol investigating the potential of baclofen in relapse prevention and its anti-craving properties in recently detoxified GHB dependent patients. Baclofen is a high affinity GABA-B receptor agonist with a half-life ranging from 2 to 6 hours [22]. The pharmacological overlap between baclofen and GHB suggests that the relatively long-acting baclofen may serve as a substitute for the short acting GHB. Moreover, baclofen is thought to modify brain reward function, through its indirect effects on dopamine, which has been suggested to be a key neurotransmitter for craving [23-25]. Finally, baclofen is thought to have anxiolytic effects through its agonist effects on the GABA-B receptor [26]. Overall, it could be speculated that baclofen may be effective in relapse prevention in GHB dependent patients. Indeed, animal data have shown beneficial effects of baclofen on GHB self-administration in mice [27].

### Aims and hypotheses

The primary aim of the current study is to assess the potential of baclofen to prevent relapse in recently detoxified GHB-dependent patients. We hypothesize that administration of baclofen to GHB-dependent patients after detoxification is associated with decreased relapse rates as compared to treatment as usual (without baclofen). We also hypothesize that treatment with baclofen is associated with reduced levels of craving for GHB and reduced psychiatric symptoms levels, including anxiety, and increased quality of life. We expect baclofen treatment to cause minimal side effects in these patients. Finally, we expect a lower drop-out rate from adjuvant therapy (TAU) in the intervention group.

## Methods and Design

### Study design

The design is of an open label non-randomized controlled clinical study in six addiction care facilities in the Netherlands. The study is part of the Dutch national GHB Monitor 2.0 and data collection will take place between May 2014 and December 2015. After successful detoxification of GHB, patients will receive either baclofen on top of treatment as usual (TAU + baclofen) or treatment as usual (TAU) only. Assignment is based on in- and exclusion criteria and on patient preference (informed consent).

### Ethical considerations

The study was approved by the Medical Ethics Committee, Twente Medical School, Institute for Applied Scientific Research (METC/14015.kam) study number NL40321.044.13. Participants are informed about the trial and about the voluntary nature of their participation with both written and verbal communications. Participants are only included following the provision of informed consent.

### Participants

#### Recruitment

Participants are GHB dependent patients in treatment for detoxification at six participating addiction care facilities. These GHB dependent patients will be informed by their physician about the possibility to participate in the current study before GHB detoxification. The physician will inform the research nurse per centre on potential participants for the study, who will provide further study information to the patients after the detoxification. After informed consent, a research physician will perform the medical screening. The intervention group will be compared with two control groups: recently detoxified GHB dependent patients included in the Dutch national GHB Monitor 2.0, but that do not want to use baclofen (TAU only); and a matched historical control group from our previous work on GHB dependence (n = 274) of whom follow-up data are available [20]. The historical control group will be matched on age, gender, GHB use (dose and years), and number of detoxification attempts.

#### In- and exclusion criteria

Patients are eligible to participate if GHB dependence (according to the DSM-IV general criteria of dependence) is their primary diagnosis [28] and their age is at least 18 years. All participants should be able to read and speak the Dutch language. Patients with any current somatic or psychiatric safety concerns are excluded. Exclusion criteria are liver cirrhosis and impaired renal function (as indicated by aspartate aminotransferase (AST)), alanine transaminase (ALT), or gamma-glutamyl transferase ((GGT) level >3 times the upper limit of normal (ULN); bilirubin > ULN; serum creatinine > ULN), unstable hypertension, unstable diabetes mellitus, seizure disorder including patients currently taking anticonvulsants, and pregnancy. Patients experiencing current severe mood disorder (bipolar disorder or major depressive disorder), current psychotic disorder (including schizophrenia), and/or suicidal ideations and patients suffering Parkinson’s disease will be excluded too. Co-current use of anxiolytics, stimulants or hypnotics will not be permitted.

#### Sample size calculation

To date, there are no published studies on the effect of baclofen on relapse in GHB dependent patients. Therefore, we used the results of previous studies on the effect of baclofen in alcohol dependent patients and our previous work on GHB dependence, in order to estimate the required sample size. Two RCTs showed that baclofen was superior to placebo in increasing abstinence rates in alcohol dependent patients: 70% (14 out of 20) versus 21% (4 out of 19) within a period of 30 days [29] and 71% (30 out of 42 p) versus 29% (14 out of 42) within a period of 12 weeks [30]. These results indicate a potential increase in abstinence of 42–49% with baclofen treatment for alcohol dependence. Results of the previous national GHB project stated that 36% of the patients succeeded to avoid relapse in GHB within a period of 3 months after detoxification without any medical interventions [20]. Therefore, we will consider a percentage of importance difference in baclofen effect in the GHB dependent participants of 34% between the Baclofen + TAU group and the TAU only or the matched control group with an expected abstinence rate with baclofen of 70% as in the alcohol studies and of 36% without baclofen.

We used the following formula comparing two proportions to calculate the sample size, n = [(Zα/2 + Zβ)^2^ × {(p1 (1-p1) + (p2 (1-p2))}] / (p1 - p2)^2^, n = [(1.96+ 0.84)^2^ × {(0.7 (1–0.7) + (0.36 (1–0.36))}] / (0.34)^2^, according to Dr. Steve Brooks sample size calculator, Exeter Initiative for Statistics. The calculation estimates that the minimal total samples size of 30 patients per group would be sufficient to detect a clinical difference of 34% in two-tailed z-test of proportions (α = 0.05, β=0.80). In the alcohol studies and our previous work with GHB inpatients an attrition rate of 13–15% was reported. We anticipate a slightly higher drop-out rate of 25%, due to the outpatient component of the baclofen treatment. Therefore we will include 40 participants in the baclofen treatment group.

### Study intervention

#### Baclofen intervention

Clinical trials on baclofen for alcohol dependence treatment have used baclofen at a dose of 30 mg ⁄d [29-31] over 30 to 120 days. This is within the low therapeutic range for muscle spasms. However, Garbutt and colleague’s [32] suggested that 30 mg of baclofen per day may be an insufficient dose for some patients to achieve abstinence. Additionally, baclofen effectiveness for GHB dependence could be smaller, since tolerance may occur due to recent abuse of GHB and a possible substitution function (as treatment will be started immediately after detoxification).

Based on these results and the unpublished results of a small dose finding experimental treatment study in our treatment center, where patients reported limited effect of baclofen 30 mg per day, we decided to administrate a dose of 45 mg to a maximal dose of 60 mg per day orally to avoid the risk of (co) intoxication. In this study baclofen will be administrated orally three times daily as usually recommended [22] and started with a total dose of 15 mg per day. During the first 10 days baclofen will be gradually increased with 15 mg per day every 3 days up to the chosen minimum dose of 45 mg, or a maximum dose of 60 mg in case no effect is reported at 45 mg after 2 weeks. This dose will subsequently be maintained for a period of 10 weeks. Successively, baclofen will be tapered off to 0 mg in 2 weeks (see Table 1). Patients will be asked to avoid abrupt termination of baclofen and will be guided by their physician to avoid complications of baclofen withdrawal. Patients who wished to continue the baclofen treatment will be offered an outpatient counselling and medication by their physician for another 3 months.

**Table 1:** **Baclofen dose in mg/week**

**Week**	**1**	**2**	**3**	**4**	**5**	**6**	**7**	**8**	**9**	**10**	**11**	**12**	**13**	**14**	**15**
Doses a day	15–30	45–60	45/60	45/60	45/60	45/60	45/60	45/60	45/60	45/60	45/60	45/60	30–45	15–30	15–0

Compliance will be assessed based on self-report, urine test for GHB, and empty pill counts. The physician will make use of the BRENDA method, a psychosocial program designed to enhance medication and treatment compliance [33,34]. In case of GHB use during baclofen treatment, participants are expected to contact the research physician. In case of relapse, immediate cessation of treatment will be considered to avoid intoxication hazards.

#### Treatment as usual (TAU)

Patients included in the current study will undergo the usual treatment provided by their addiction treatment center (TAU). These can vary from short individual behavioral treatment intervention, inpatient treatment, Community reinforcement therapy, or extensive multidimensional therapy (like MDFT) with attention to psychological, social, relation, financial, and medical problems. During the study the psychological interventions applied will be monitored. For the historical control group, reports on the adjunct psychological treatment interventions applied are unavailable.

### Outcome and instruments

#### Primary outcome measures

The primary study outcome is the relapse rate, in other words the level of GHB use as indexed by the total number of abstinent days, the duration of continued abstinence after detoxification (CAD), time before relapse, and intensity of substance use over a period of 3 and 6 months (Timeline Follow-back Method).

#### Secondary outcome measures

The secondary outcomes as listed below are;

The craving level, as indexed by self-report using the Desire for Drugs Questionnaire (DDQ) and a visual analogue scale (VAS). The DDQ is validated instrument [35] which measures an instant desire, triggered by internal or external cures (instant craving). Franken and colleagues [35] identified three factors as underlying dimensions, namely ‘desire and intention’ (seven items), ‘negative reinforcement’ (four items), and ‘control’ (two items) with a scale from 1 (totally disagree) to 7 (totally agree). The total score exists of 14 items with a sum score between 14 and 98 [35]. In addition to that patients were asked to rate craving for GHB by means of a visual analogue scale (VAS) on a Vertical line from no craving at all (at the bottom) with a score of 0, to extremely strong craving (at the Top) with a score of 100. The result is a score on a continuous scale ranging from 0 to 100.

A change in psychiatric symptoms will be measured by means of the Mini International Neuropsychiatric Interview-plus (MINI-plus) and Depression Anxiety Stress scale (DASS). A trained therapist or physician will apply the MINI-plus. It is a structured interview to assess the main Axis I psychiatric disorders based upon the DSM–IV and ICD-10 criteria. It is used to determine current and lifetime psychiatric disorders [36]. The MINI is shown to have good psychometric properties and is reliable for the detection and classification of psychiatric comorbidity [37]. The DASS self-report will be used to measure psychiatric symptom levels and the related negative emotional states along the 3 axes of depression, anxiety and stress. It is a 21-item self-report instrument. Participants are asked to use a 4-point severity scales to rate the extent to which they have experienced each state over the past week. Scores for Depression, Anxiety and Stress are calculated by summing the scores for the relevant items. For each scale threshold values are proposed and are interpreted in 5 severity intensities: normal, mild, moderate, severe and extremely severe. Thresholds as (for Depression ≥ 21; for Anxiety ≥15; for Stress ≥ 26) indicate a severe or extremely severe distress state [38,39].

The EuroQol-5D (EQ-5D) will be used to measure changes in patients’ health-related quality of life. For the overall quantification of health status as a single index we will use the standard EQ-5D classification system developed by the EuroQol Group [40]. The EQ-5D is a widely used multi-attribute system available to determine health state preferences (utilities). It is a simple self-report instrument which assesses 5 domains of general health and functioning: mobility, self-care, usual activities, pain/discomfort and anxiety/depression. This is along a 3-point scale (1 = no problems, 2 = some problems and 3 = extreme problems). Based on the descriptive classification of the EQ-5D system a preference index (utility) can be estimated that expresses the overall preference of the classified health status [41] [the Netherlands algorithm will be used to calculate the index]. This EQ-5D index measures objective quality of life and is a societal-based numerical quantification of the patients’ health status in a scale from-.594 to 1 (perfect health), 0 is (as bad as being dead). In addition, participants will be asked to rate the overall health related quality of life, by means of a visual analogue scale (EQ-5D VAS). The VAS is a 100-mm vertical line from worst (0) to optimal of health (100). The EQ-5D VAS represents the subjective quality of life.

Safety of baclofen will be assessed by the number and intensity of reported side effects, using weekly report of adverse effects on a checklist and standardized medical assessments. The side-effects checklist is based on published side effects of baclofen in the treatment of Multiple Sclerosis [42]. Side-effects are partly self-monitored (21 items) and partly observed (8 items) by a doctor or nurse practitioner. Every item is scored on a five-point Likert scale: never (0), seldom (1), sometimes (2), frequently (3) or always (4). Examples of items are vomiting, nausea and diarrhea. The checklist also contains 5 parameters measured by a doctor or nurse practitioner. Examples of parameters are body temperature and heart rate frequency.

#### Additional measures

Addiction physicians will be asked to monitor withdrawal symptoms when tapering baclofen, using standard questions. These include a short check of the most reported withdrawal symptoms in the literature [43,44].

We will monitor the use of other substances over the last thirty days (number of days and quantity) using section 1 of the Measurement of Addicts for Triage and Evaluation (MATE) before and after detoxification and at follow up (3 months). The MATE is a Dutch instrument designed as an aid in the diagnosis of clinically relevant patient characteristics in substance use disorders according to the DSM-IV axes [45].

At 6 month period we will also report on the needed period of treatment with baclofen to maintain abstinence by monitoring relapse (Timeline Follow back) and craving assessment (DDQ, VAS) within the intervention group between those who used baclofen for 3 month only and those who choose to continue the treatment further.

### Procedure and data collection

Patients in all treatment conditions (baclofen + TAU or TAU only) will be assessed by research assistants identically in the following time points. Before detoxification, the intensity of GHB abuse (MATE), Quality of life (EQ-5D) and psychiatric comorbidity (DASS) will be assessed. At the end of the detoxification treatment (baseline) and at follow up (three months after start of detoxification) craving (VAS, DDQ), psychiatric comorbidity (MINI plus, DASS), and Quality of life (EQ-5D) will be assessed.

The addiction physician will monitor the participants in the baclofen condition during regular medical check-ups for potential side effects (side effect checklist) and craving (VAS, DDQ), see Table 2. During the follow-up (six months after start of detoxification) the DDQ, DASS, MINI-plus, EQ-5D will be repeated. In summery self-report questionnaires will be administered at the start of detoxification; start of baclofen treatment (week 0), baclofen titration and stabilization (week 1 + 2) and maintenance treatment (week 3–12). Add to that, three months after the end of the baclofen treatment, a follow-up measurements will take place. The research assistant will build up an online Client Record Form from all the questionnaires filled in by the patients. A summary of the medical records will be added by the physician to the electronic patient file.

**Table 2:** **Time measurements**

**Week**	**0**	**1**	**2**	**3**	**4**	**5**	**6**	**7**	**8**	**9**	**10**	**11**	**12**	**13**	**14**	**15**	**24**
Measurement	**T0**-baseline	**T1**											**T2**				**T3**
Mate section	xx												xx				x
EQ–5D	xx												xx				x
DASS	xx												xx				x
Mini-plus	xx												xx				x
Blood	xx						x						x				
Urine	xx						x						xx				x
Medical consult	2x	2x	2x	x	x			x			x		x	x	x		
Baclofen (mg)	0	15–30	45– 60											30–45	30–15	15–0	0
Baclofen Side effect	x	2x	2x	x	x			x			x		x				
VAS	xx	x	x	x	x			x			x		xx				x
DDQ	xx	x	x	x	x			x			x		xx				x
Baclofen withdrawal checklist														x	x	x	
Timeline follow back													xx				x

xx = baclofen group and control group TAU only.

x = baclofen group.

T0 = end of GHB detoxification.

T1 = start baclofen treatment.

T2 = follow-up TAU only and end of baclofen therapy.

T3 = follow-up baclofen group.

### Statistical analyses

Descriptive analysis will be executed for all measurements and will include the mean, SD and frequencies of events with confidence intervals. Descriptive statistics will be used to compare the basic characteristics of the participants in the experimental group and the control groups. To assess any difference in relapse rates between the experimental group and the control groups (TAU group and matched historical group) at 3 month after detoxification Pearson Chi-squared test will be used for the dichotomous abstinence rates. ANOVA will be carried out on continuous variables, such as the total number of abstinent days, the maximum duration of continued abstinence, time before relapse (relapse defined as ≥ 3 times GHB use per day, for 2 subsequent days), and level of substance use over a period of 3 months. At 6 months period participants who continued baclofen will be compared to those who have stopped with baclofen (after 3 month) on relapse into GHB abuse or abstinence corrected for craving. The numbers of patients maintaining abstinence will be analysed with the intention-to-treat principles. The difference in craving will be analysed by MANCOVA at 3 months between groups (abstinent experimental versus abstinent TAU or relapsed TAU) with DDQ and VAS craving as dependent variables and corrected for craving levels immediately after detoxification. The change in craving in time during the baclofen treatment will be analysed via repeated measures MANOVA. By means of MANCOVA at 6 months after detoxification the difference in craving among participants who are still using baclofen and those who have discontinued baclofen will be analysed corrected for craving after 3 months. The effect of baclofen on the psychiatric symptoms levels (DASS, MINI) and also quality of life (EQ–5D) will be analyzed similarly as for craving at 3 and 6 months. The effects of the different TAU approaches as potential confounders will be tested in a sensitivity analyses (changing one-factor-at-a-time) in an linear regression including TAU per center as covariates.

Safety of baclofen, as assessed by the number and intensity of reported side effects, will be analyzed in the following categories: acceptable or unacceptable adverse effect, clinically significant deterioration, and relocation. In all analyses socio-demographic characteristics such as age and gender will be considered as co-variates in the analysis. Two-sided p-value of > .05 is considered statistically significant. The statistical software package SPSS will be used for all the computations.

## Discussion

This study protocol presents the design of an open label clinical trial evaluating the efficacy of baclofen to prevent relapse, reduce craving and anxiety, and to assess the safety profile of baclofen in GHB dependent patients. To date, there are no reports on the potential of baclofen to prevent relapse in GHB dependent patients. We expect that baclofen will increase abstinence rates, reduce craving and will be well tolerated. In addition, baclofen may improve psychiatric symptoms such as anxiety and depression, as suggested in several clinical trials in alcohol-dependent individuals [29,30,46].

Several risks of baclofen use are taken into account in the current study. In patients with neuropsychiatric disorders, such as schizophrenia, severe depression, mania, Parkinson’s disease and cerebrovascular diseases, exacerbations of these conditions may occur, when the dose of baclofen is rapidly increased. It may lower the threshold for seizures in epileptic patients [42]. To limit these risks in the current trial, baclofen will be uploaded gradually, over a period of minimally 10 days. Moreover, patients previously diagnosed with any of the mentioned diseases will be excluded from the study.

Abrupt discontinuation of baclofen, when used for several months, can be associated with a withdrawal syndrome which resembles benzodiazepine and alcohol withdrawal. Several symptoms are reported such as hallucinations, fever, confusion, delirium, agitation, insomnia, and muscle stiffness and spasms [47]. Therefore, patients will be advised to taper down slowly when discontinuation is needed.

Intoxication with baclofen has been reported in doses above 100 mg [48]. This risk increases if patients combine baclofen with GHB. Intoxication is not expected when baclofen is administered at a low dose (<30 mg per gift), as applied in the current study. Patients will however, be informed repeatedly of this potential risk and the treatment will be discontinued immediately in case of relapse.

The current study does have several limitations. First, the study is not randomized, nor placebo controlled. However, we will compare patients receiving baclofen with a control group matched for gender, age and the pattern of GHB use, in order to control for potential confounding factors. Second, the accompanying treatment as usual (TAU) is not identical in all participating addiction care facilities. This may confound our results. The impact of TAU will be checked with a sensitivity analysis compare patients’ results from the different centers conforming the add-on baclofen effect.

Given these methodological limitations the current study should be considered explorative in nature. Given the currently rather limited information from both preclinical and clinical studies of GHB such an explorative approach seems justified. We would also like to test the GHB substitution effect of baclofen (as a GABA-B agonist) hypothesis. It is important to evaluate of this effect is clinically recognized and obvious for the participants, and may define placebo use as non-beneficial. We need to be able to determine the margins of the required treatment dose. All forgoing justify this setup of an open label trail before a large placebo controlled randomized trial.

### Implications for practice

If our study confirms the potential of baclofen to reduce relapse and craving without serious adverse events in GHB-dependent patients, this warrants large scale randomized controlled trials in order to draw more firm conclusions. If baclofen showed to have beneficial effects on psychiatric symptoms in these patients, baclofen might be specific interest for the treatment of those GHB dependent patients with co-morbid high levels of anxiety and depression.
